# Dietary Cocoa Flavanols Do Not Alter Brain Excitability in Young Healthy Adults

**DOI:** 10.3390/nu16070969

**Published:** 2024-03-27

**Authors:** Raphael Hamel, Rebecca Oyler, Evie Harms, Rosamond Bailey, Catarina Rendeiro, Ned Jenkinson

**Affiliations:** 1 School of Sports, Exercise and Rehabilitation Sciences, University of Birmingham, Birmingham B15 2TT, UK; 2 Centre for Human Brain Health, School of Psychology, University of Birmingham, Birmingham B15 2TT, UK

**Keywords:** cocoa flavanols, paired-pulse transcranial magnetic stimulation (ppTMS), brain excitability, corticospinal excitability (CSE), corticospinal silent period (CSP), short intracortical facilitation (SICF), short intracortical inhibition (SICI), intracortical facilitation (ICF), long intracortical inhibition (LICI)

## Abstract

The ingestion of dietary cocoa flavanols acutely alters functions of the cerebral endothelium, but whether the effects of flavanols permeate beyond this to alter other brain functions remains unclear. Based on converging evidence, this work tested the hypothesis that cocoa flavanols would alter brain excitability in young healthy adults. In a randomised, cross-over, double-blinded, placebo-controlled design, transcranial magnetic stimulation was used to assess corticospinal and intracortical excitability before as well as 1 and 2 h post-ingestion of a beverage containing either high (695 mg flavanols, 150 mg (−)-epicatechin) or low levels (5 mg flavanols, 0 mg (−)-epicatechin) of cocoa flavanols. In addition to this acute intervention, the effects of a short-term chronic intervention where the same cocoa flavanol doses were ingested once a day for 5 consecutive days were also investigated. For both the acute and chronic interventions, the results revealed no robust alteration in corticospinal or intracortical excitability. One possibility is that cocoa flavanols yield no net effect on brain excitability, but predominantly alter functions of the cerebral endothelium in young healthy adults. Future studies should increase intervention durations to maximize the acute and chronic accumulation of flavanols in the brain, and further investigate if cocoa flavanols would be more effective at altering brain excitability in older adults and clinical populations than in younger adults.

## 1. Introduction

Observational studies suggest that a high intake of dietary flavonoids results in improved cognitive evolution later in life [1,2,3,4]. In addition to their capacity to alleviate neuroinflammation [5,6], flavonoids have also been suggested to enhance cognition by improving the regulation of blood flow to the brain [7,8,9,10,11]. Namely, in healthy adults, the ingestion of a single dose of cocoa flavanols acutely improves cognitive performance at counting backwards in steps of three [12], as well as at visually identifying target items [13] and motion direction [14] (see [15,16] for meta-analyses). Further work has associated these cognitive enhancements with the capacity of cocoa flavanols to acutely increase cortical blood perfusion [7,11], cortical oxygenation [8,9], and neurovascular coupling [10], suggesting that flavanols improve cognitive performance through their vascular effects. Interestingly, converging lines of evidence now suggest that cocoa flavanols also alter brain excitability (see below), implying that the effects of flavanols permeate beyond the cerebral endothelium. This possibility is compelling and important to address, as it could reveal novel mechanisms by which flavanols alter brain health and cognition. The overarching objective of this study was to examine whether cocoa flavanols alter brain excitability, which is supported by at least two possibilities.

A first possibility is that cocoa flavanols cross the brain–blood barrier (BBB) and *directly* alter brain excitability. Namely, extensive in vitro and vivo animal work shows that flavonoids—including flavanols and anthocyanins—accumulate in brain tissue following chronic intake [17,18,19,20,21,22,23] and can also cross the BBB in a relatively short timeframe (~30 min to 2 h after ingestion) [24,25,26,27,28,29]. This evidence is noteworthy, as it suggests that flavanols can quickly diffuse beyond the cerebral endothelium. However, whether flavanols acutely cross the BBB in the human brain remains unclear [30,31]. Nonetheless, the chronic intake of flavonoids has been shown to result in the accumulation of flavonoid-derived gut metabolites (e.g., caffeic acid) in human cerebrospinal fluid [22,32], suggesting that flavanols diffuse beyond the human BBB and reach brain tissue. Once in the brain, converging evidence suggests that cocoa flavanols directly alter excitability by influencing glutamatergic and gamma aminobutyric acid (GABA)ergic transmission. Namely, in vitro work has shown that flavonoids decrease neuronal firing rates [33], GABAergic inhibition [34,35], and the activity of GABA and glutamate transporters [36], suggesting that flavanols alter neuronal excitability. In addition, animal evidence further suggests that chronic interventions with flavonoid-rich foods also modulate synaptic plasticity [37], presumably by altering the activity of glutamatergic [38] and GABAergic receptors [39], amongst other mechanisms [40]. Interestingly, recent evidence suggests that such changes in excitability can also be observed in humans. Indeed, human work has recently shown that indicaxanthin—a phytochemical extracted from the Opuntia Ficus Indica cactus—acutely increases intracortical excitability [41]. Overall, this evidence suggests that phytochemicals such as cocoa flavanols cross the BBB and *directly* alter brain excitability.

Also known as the haemo-neural hypothesis (see [42] for a review), another possibility is that cocoa flavanols do not need to cross the BBB to alter brain excitability, but can *indirectly* do so by modulating neurovascular coupling. Namely, cocoa flavanols induce vasodilation by causing the release of nitric oxide (NO) [43,44,45,46,47], which is believed to account for their neurovascular coupling enhancements [7,8,48]. To indirectly alter brain excitability, one proposed mechanism is that the endothelium-borne NO would diffuse beyond the BBB [49,50,51,52] to modulate neuronal excitability [53,54,55,56], notably by altering axonal excitability [57], activity at glutamatergic synapses [58] and the release of GABA [59]. Another proposed mechanism is that the cocoa flavanol-induced vasodilation causes mechanical forces that would modulate brain excitability through mechanosensitive ion channels [60] including astrocytic TRPV4 channels [61], but also through type B GABA (GABA_B_) and adenosine A_1_ receptors [61]. Overall, this evidence suggests that cocoa flavanols *indirectly* alter brain excitability through neurovasculature-mediated mechanisms.

Based on this background evidence, this work set out to test the hypothesis that cocoa flavanols would alter brain excitability in humans. In a randomised, cross-over, double-blind design, corticospinal and intracortical excitability was assessed before as well as 1 and 2 h after participants ingested a beverage containing either high or low levels of cocoa flavanols. To achieve this, single and paired-pulse transcranial magnetic stimulation (TMS) was applied over the left motor cortex (M1) to assess brain excitability [62]. Specifically, TMS was used to evaluate corticospinal excitability and inhibition, as well as intracortical facilitation and inhibition, which can be used to infer changes in glutamatergic and GABAergic activity [62]. Finally, based on evidence suggesting that polyphenols require repeated dosing to accumulate in brain tissue [17,18,19,20,21,22,23], the short-term chronic effects of ingesting cocoa flavanols for 5 consecutive days on brain excitability were also investigated in this work.

## 2. Methods

### 2.1. Participants

A total of 20 medication-free and neurologically healthy young adults gave their informed consent to participate in this study (12 females; 22 ± 1 years old; mean ± 95% CIs). Participants were screened for TMS contraindications [63]. The study conformed with the 1964 Declaration of Helsinki and was approved by the local institutional review board (ERN 18-2077APN11). Participants were offered research credits in exchange for their participation.

An a priori sample size analysis was conducted using G*Power (v3.1.9.4). The smallest effect size of interest in this study was a Cohen’s dz of 0.8 (large effect size). Assuming two-tailed dependent *t*-tests, 80% power, and alpha of 0.05, the results revealed that 15 participants would be required. However, since this study also involved between-subject comparisons, a total of 20 participants were recruited (*n* = 10 per group; see below).

### 2.2. Protocol Overview

**Figure 1:**
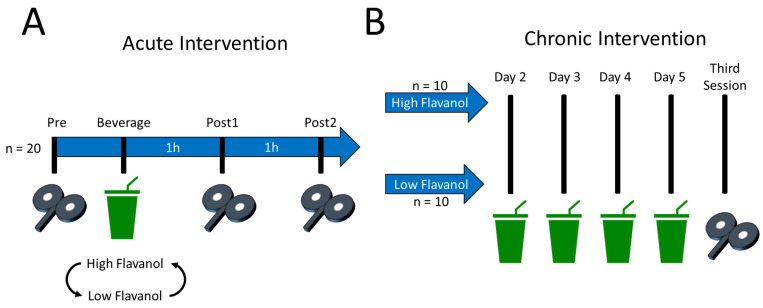
Overview of the acute and chronic interventions. (**A**) Acute intervention. All participants (*n* = 20) took part in two sessions, where they ingested either a high flavanol or low flavanol (placebo) beverage. Excitability was assessed immediately before as well as 1 (60 min) and 2 h (120 min) after beverage ingestion. (**B**) Chronic intervention. Depending on the beverage that was ingested on the second session of the acute intervention, participants were allocated to one of two groups for the chronic intervention: high flavanol (*n* = 10) and low flavanol (*n* = 10). Specifically, participants were sent back home with the instruction to drink one high- or low-flavanol beverage per day for 4 consecutive days. Thus, a total of 5 beverages were ingested for the chronic intervention. In a third session, the effects of the chronic intervention on brain excitability were then assessed. All procedures were double-blinded.

All procedures were randomised, crossed-over, double-blinded and placebo-controlled. An overview of the protocol is shown in Figure 1. Globally, the procedures were within-subject for the acute intervention and became between-subject for the chronic intervention. All participants executed the acute intervention before the chronic one. To enhance the ecological validity of the design, all participants were asked to maintain their dietary habits, exercise levels, and caffeine consumption 24 h before each session for both the acute and chronic interventions. It was reasoned that the effects of flavanols on brain excitability should be apparent regardless of participants’ habits for the results to yield generalisable implications.

For the acute intervention, participants first took part in two sessions in which the acute effects of the high- and low-flavanol beverages on corticospinal and intracortical excitability were assessed (Figure 1A). Hereafter, these two sessions are referred to as “high flavanol” and “low flavanol”. The sessions of the acute intervention were counterbalanced, took place at the same time of day for a given participant, and were separated by an average of 20 ± 8 days. The second session of this acute intervention constituted the first day of a 5-day chronic intervention. Namely, upon completion of the acute intervention, participants were sent back home with a 4-day supply of high- or low-flavanol beverages, whichever they last ingested on their second session. For the chronic intervention, the 20 participants were thus separated into two equal groups of 10 participants, hereafter referred to as the “high-flavanol group” and “low-flavanol group” (Figure 1B). Participants were instructed to ingest their beverage at the same time of day as during the acute intervention. Finally, participants came back for a third session to assess the chronic effects of cocoa flavanols on corticospinal and intracortical excitability.

### 2.3. Preparation and Composition of the Cocoa Flavanol Interventions

For the acute intervention, the beverages were prepared immediately before consumption by dissolving 12 g of cocoa powder in 300 mL of room-temperature Buxton bottled water, which contains low levels of nitroso species. The beverages were delivered to participants in an opaque container and were drunk with an opaque straw. For the chronic intervention, participants were instructed to dissolve the cocoa powder in 300 mL of tap water. The cocoa powders used were commercially available (manufactured by Barry Callebaut, Zürich, Switzerland): the high-flavanol cocoa powder was a non-alkalised fat-reduced powder (“Natural Acticoa”) and the low-flavanol control was a fat-reduced alkalised cocoa powder (commercial name: 10/12 DDP Royal Dutch). Both beverages were matched for texture, consistency, and taste (see [8,64]), as well as macronutrient and micronutrient content, including both caffeine and theobromine (see Table 1 for details). Importantly, the high-flavanol intervention delivered 150 mg of (−)-epicatechin and 85.4 mg of (−) and (+) catechin monomers, whilst the low-flavanol intervention delivered < 6 mg of both monomers. These flavanol monomer doses were based on previous studies showing that they robustly modulate acute human endothelial functions [46,65,66] and plasma nitric oxide levels [45]. Moreover, similar monomer doses (e.g., (−)-epicatechin) can be achieved through diet by consuming foods rich in flavanols [67], suggesting that the present flavanol doses have ecological validity.

The total levels of polyphenols in the present cocoa powders were assessed by a Folin–Ciocâlteu reagent calorimetric assay (as described in [68]). The individual monomer, procyanidin, and methylxanthine levels were confirmed by high-performance liquid chromatography (HPLC; as described in [69,70]). The individual doses of cocoa powder were identified by a three-digit code to ensure double-blindness. For the acute intervention, doses were kept at −20 °C. For the chronic intervention, participants were instructed to keep the cocoa powder doses in their freezer. Finally, participants and researchers involved in data collection and analysis were blinded to the intervention conditions until all data analysis was completed.

### 2.4. Acute Intervention: Justification of the TMS Time Points

For the acute intervention only, corticospinal and intracortical excitability was measured immediately before as well as 1 and 2 h after beverage ingestion (Figure 1A). Hereafter, these time points are referred to as Pre, Post1, and Post2, respectively. This was based on prior human studies showing that flavanol monomer metabolites require ~2 h to reach maximum systemic concentrations [71], as well as to enhance cognitive functions and cerebral oxygenation [8]. In the acute intervention, it was thus expected that dietary flavanols would alter brain excitability ~2 h following beverage ingestion.

### 2.5. EMG and Neuronavigated TMS

Electromyography (EMG) data from the right first dorsal interosseus (FDI) muscle belly were recorded through a single bipolar electrode connected to a 2-channel Delsys Bagnoli (Delsys^®^, Natick, MA, USA) system, itself connected to a Micro 1401 data acquisition unit (Cambridge Electronic Design, Cambridge, UK). The EMG data were acquired with Signal (Cambridge Electronic Design, v6.05) at a sampling rate of 10,000 Hz for epochs of 600 ms (200 ms pre-trigger time). The EMG data were high- and low-pass filtered at 20 Hz and 450 Hz, respectively, with a notch at 50 Hz. The reference EMG electrode was positioned on the proximal olecranon process of the right ulnar bone. The EMG data were analysed using an automated custom-built MATLAB (R2022b, Mathworks^®^, Natick, MA, USA) script.

Neuronavigated TMS pulses were delivered through a single figure-of-eight 70 mm Alpha Flat Coil (taped and uncased) connected to a paired-pulse BiStim^2^ stimulator (MagStim, Whitland, UK). BrainSight (Rogue Research; Montreal, QC, Canada) was used to ensure reliable coil positioning during every experiment and session [72]. The coil was positioned at a 45° angle in a posterior–anterior axis over the FDI motor hotspot of the left M1, defined as the area where motor-evoked potentials (MEPs) of maximal amplitude could be reliably elicited with suprathreshold TMS pulses. The resting motor threshold (RMT) was defined as the percentage of maximum stimulator output to induce 5 MEPs out of 10 TMS pulses of at least 50 µV of peak-to-peak amplitude [63]. The test stimulus (TS) intensity was calibrated to obtain MEPs of ±1 mV (see TMS variables for details on parameters used). For every participant, the FDI motor hotspot, RMT, and TS intensity were assessed at the start of every session. Once determined, these parameters remained constant for a given session. In this work, the average intensities for the RMT and TS were 50 ± 3% and 60 ± 3% of the maximum stimulator output, respectively.

### 2.6. Definition of the TMS Variables

The effects of cocoa flavanols on corticospinal excitability (CSE) and corticospinal silent period (CSP) duration were measured. Namely, CSE is believed to reflect the excitability of cortical, subcortical, and spinal structures [73]. To assess CSE, single pulses of TMS at TS intensity were delivered [74]. CSP duration is believed to reflect the extent of corticospinal inhibition [73], presumably originating from GABAergic mechanisms in cortical, subcortical, and spinal structures [75]. To assess CSP duration, single pulses of TMS at TS intensity were delivered whilst participants squeezed a dynamometer with their right hand at an intensity equivalent to 30% of their maximum voluntary contraction (similar to [74]).

To evaluate the effects of cocoa flavanols on intracortical excitability, short intracortical facilitation (SICF), short intracortical inhibition (SICI), intracortical facilitation (ICF), and long intracortical inhibition (LICI) were measured. All these variables were assessed by delivering paired pulses of TMS. Namely, SICF is believed to reflect intracortical glutamatergic activity [73]. To assess SICF, the first pulse was delivered at TS intensity, and the second pulse was delivered at 100% of the RMT after an interval of 3 ms [73]. SICI is believed to reflect intracortical GABA_A_-mediated inhibition [73]. To assess SICI, the first pulse was delivered at 70% of the RMT, and the second pulse was delivered at TS intensity after an interval of 3 ms [73]. Similarly to SICF, ICF is also believed to reflect intracortical glutamatergic activity [73]. To assess ICF, the first pulse was delivered at 70% of the RMT, and the second pulse was delivered at TS intensity after an interval of 15 ms. Finally, LICI is believed to reflect type B GABA (GABA_B_)-mediated inhibition [73]. To assess LICI, the first and second pulses were delivered at TS intensity, which were separated by an interval of 150 ms [73].

Given that this study is the first to evaluate the effects of cocoa flavanols on brain excitability, these 6 variables were chosen to provide a thorough range of assessments of corticospinal and intracortical excitability. To ensure robust estimations of brain excitability, a total of 30 pulses per time point were recorded for SICF, SICI, ICF, and LICI [76,77]. CSE was derived from the first pulse of LICI [74], which also resulted in 30 measurements. To assess CSP, 15 trials were recorded, which also ensures robust estimations [78]. All these variables were collected at rest with a pseudorandomised schedule, except for CSP, where an isometric contraction of the hand had to be performed. Per time point, ~20 min was required to assess these variables. As a result, to provide excitability measures at 1 and 2 h after beverage ingestion, these variables were assessed over 20 min spanning 10 min before as well as after the Post1 and Post2 time points.

### 2.7. Calculations of the TMS Variables

For CSE, SICF, SICI, ICF, and LICI, the peak-to-peak amplitude of the MEPs induced by the TS were first calculated. To isolate intracortical excitability measures, SICF, SICI, ICF, and LICI were normalised to CSE. Namely, the individual MEP amplitudes of the SICF, SICI, ICF, and LICI trials were divided by the average MEP amplitude of CSE separately for each time point and participant, resulting in a percentage change. The individual SICF, SICI, ICF, and LICI trials were then averaged separately for each time point and participant.

To calculate CSP duration, a custom-designed algorithm in MATLAB (R2022b, Mathworks^®^) was used. Namely, CSP duration was measured as the time difference (in milliseconds) between the delivery of the TMS pulse and the return of the EMG of voluntary muscle activity (CSP offset). The CSP offset was determined as the moment when the SD of a sliding window spanning 2.5 ms exceeded 50% of the SD of the EMG background activity, calculated over the 100 ms that immediately preceded TMS pulse delivery, for at least 5 ms [74,79]. The EMG data were not rectified.

For the acute intervention, the TMS variables of the Post1 and Post2 time points were normalised to the Pre time point, therefore resulting in a percentage change from baseline excitability values. For each participant and each of the two beverages, this was done separately for all 6 TMS variables. For the chronic intervention, the TMS variables of the third session were normalised to the data from the Pre time point of the second acute intervention, also resulting in a percentage change from baseline excitability values. This was also done separately for each participant and all 6 TMS variables. Hereafter, the second and third sessions of the chronic intervention are referred to as pre-chronic and post-chronic, respectively (Figure 1B).

### 2.8. Statistical Analyses

To analyse the results, repeated-measures analyses of variance (ANOVAs) were conducted. For the acute intervention, the within-subject factors were beverages (high flavanol, low flavanol) and time points (Pre, Post1, Post2). For the chronic intervention, the between-subject factors were groups (high flavanol, low flavanol) and sessions (pre-chronic, post-chronic). If the data violated the assumptions of sphericity (*p* < 0.05, Mauchly test), the Greenhouse–Geiser correction was applied. If data deviated from normality upon pairwise comparisons (*p* < 0.05; Shapiro–Wilk test), non-parametric pairwise comparisons were conducted (Wilcoxon rank test rather than dependent *t*-test for within-subject comparisons; Mann–Whitney U test rather than independent *t*-test for between-subject comparisons). The Benjamini–Hochberg correction [80] was used to control for inflated type 1 error upon multiple comparisons. The statistical significance threshold was set at 0.05. All descriptive statistics reported in this work represent the means ± 95% CIs. The open-access software JAMOVI (v2.3.28) was used to conduct the statistical analyses.

### 2.9. Adverse Event Report

None of the participants reported adverse events for the acute or chronic interventions.

## 3. Results

### 3.1. Acute Intervention

#### 3.1.1. No Acute Effects of Cocoa Flavanols on Corticospinal Excitability or Inhibition

**Figure 2:**
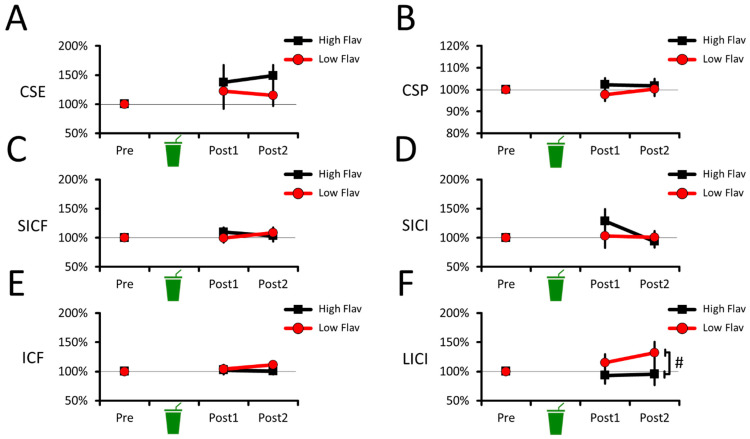
Corticospinal and intracortical excitability changes during the acute intervention. No difference between beverages was observed in CSE (**A**), CSP (**B**), SICF (**C**), SICI (**D**), or ICF data (**E**). For LICI data (**F**), a main effect of beverage was observed, where the low-flavanol beverage marginally decreased LICI compared to the high-flavanol one. For all panels, the means and 95% CIs of each condition are shown. # Marginally significant difference (*p* < 0.1, but *p* > 0.05).

The CSE data (Figure 2A) revealed no effect of beverages (F_(1,19)_ = 1.704, *p* = 0.207, ${ƞ}_{p}^{2}$ = 0.082) or beverage–time point interaction (F_(2,38)_ = 0.689, *p* = 0.469, ${ƞ}_{p}^{2}$ = 0.035), but revealed an effect of time points (F_(2,38)_ = 4.428, *p* = 0.019, ${ƞ}_{p}^{2}$ = 0.189). For time points, however, none of the pairwise comparisons survived multiple comparisons. Namely, CSE marginally increased at Post1 (130 ± 12%; *p* = 0.098, Cohen’s dz = 0.526) and at Post2 (132 ± 14%; *p* = 0.104, Cohen’s dz = 0.509) compared to Pre (100%). CSE at Post1 did not differ from Post2 (*p* = 0.805, Cohen’s dz = 0.056). This shows that CSE did not differ between the high- and low-flavanol beverages, suggesting no acute change in corticospinal excitability.

Similarly, the CSP data (Figure 2B) revealed no effect of beverages (F_(1,19)_ = 1.344, *p* = 0.261, ${ƞ}_{p}^{2}$ = 0.066), no effect of time points (F_(2,38)_ = 0.286, *p* = 0.753, ${ƞ}_{p}^{2}$ = 0.015), and no beverage–time point interaction (F_(2,38)_ = 0.952, *p* = 0.395, ${ƞ}_{p}^{2}$ = 0.048). This shows that CSP duration did not differ between the high- and low-flavanol beverages, suggesting no acute change in corticospinal inhibition.

#### 3.1.2. No Acute Effects of Cocoa Flavanols on Intracortical Facilitation or Inhibition

The SICF data (Figure 2C) revealed no effect of beverages (F_(1,19)_ = 0.134, *p* = 0.719, ${ƞ}_{p}^{2}$ = 0.007), no effect of time points (F_(2,38)_ = 0.884, *p* = 0.421, ${ƞ}_{p}^{2}$ = 0.045), and no beverage–time point interaction (F_(2,38)_ = 1.259, *p* = 0.295, ${ƞ}_{p}^{2}$ = 0.062). This shows that SICF did not differ between the high- and low-flavanol beverages, suggesting no acute change in intracortical facilitation.

The SICI data (Figure 2D) revealed no effect of beverages (F_(1,19)_ = 0.425, *p* = 0.522, ${ƞ}_{p}^{2}$ = 0.022) and no beverage–time point interaction (F_(2,38)_ = 1.804, *p* = 0.190, ${ƞ}_{p}^{2}$ = 0.087), but revealed an effect of time points (F_(2,38)_ = 3.362, *p* = 0.053, ${ƞ}_{p}^{2}$ = 0.150). For time points, however, none of the pairwise comparisons survived multiple comparisons. Namely, SICI tended to decrease at Post1 (116 ± 14%; *p* = 0.113, Cohen’s dz = 0.500) compared to Pre (100%). SICI at Post2 (97% ± 13%) marginally increased compared to Post1 (*p* = 0.084, Cohen’s dz = 0.455), but did not differ from Pre (*p* = 0.694, Cohen’s dz = 0.089). This shows that SICI did not differ between the high- and low-flavanol beverages, suggesting no acute change in intracortical inhibition.

The ICF data (Figure 2E) revealed no effect of beverages (F_(1,19)_ = 1.170, *p* = 0.293, ${ƞ}_{p}^{2}$ = 0.058), no effect of time points (F_(2,38)_ = 0.927, *p* = 0.384, ${ƞ}_{p}^{2}$ = 0.047), and no beverage–time point interaction (F_(2,38)_ = 1.379, *p* = 0.264, ${ƞ}_{p}^{2}$ = 0.068). This shows that ICF did not differ between the high- and low-flavanol beverages, suggesting no acute change in intracortical facilitation.

The LICI data (Figure 2F) revealed an effect of beverages (F_(1,19)_ = 4.886, *p* = 0.039, ${ƞ}_{p}^{2}$ = 0.205), but no effect of time points (F_(2,38)_ = 0.931, *p* = 0.403, ${ƞ}_{p}^{2}$ = 0.047) and no beverage–time point interaction (F_(2,38)_ = 1.976, *p* = 0.153, ${ƞ}_{p}^{2}$ = 0.094). For beverages, LICI marginally decreased for low flavanol (116% ± 17%) compared to high flavanol (96% ± 17%; *p* = 0.058, Cohen’s dz = 0.494). However, neither high (*p* = 0.506, Cohen’s dz = 0.152) nor low flavanol (*p* = 0.161, Cohen’s dz = 0.413) differed from pre-intervention values (100%). Overall, this shows that low flavanol acutely decreased LICI compared to high flavanol. However, the absence of beverage–time point interactions and lack of difference from pre-intervention values make it unclear if the high-flavanol beverage robustly altered LICI compared to the low-flavanol one.

### 3.2. Chronic Intervention

#### 3.2.1. No Chronic Effects of Cocoa Flavanols on Corticospinal Excitability or Inhibition

**Figure 3:**
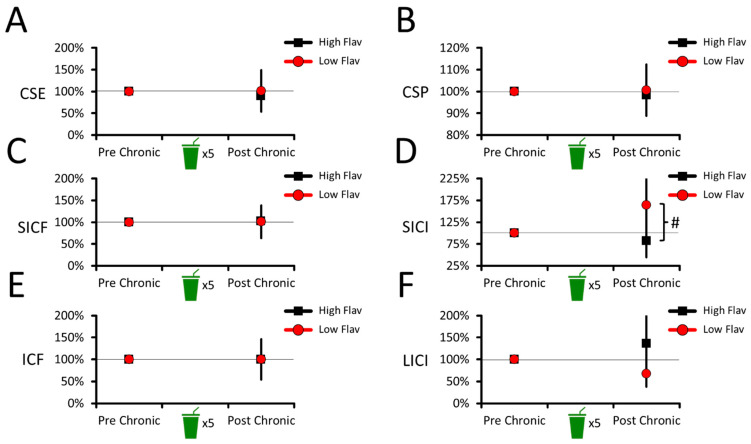
Corticospinal and intracortical excitability changes during the chronic intervention. No difference between beverages was observed in CSE (**A**), CSP (**B**), SICF (**C**), ICF (**E**), or LICI data (**F**). For SICI data (**D**), the results revealed that the low-flavanol beverage marginally decreased SICI compared to the high-flavanol one. For all panels, the means and 95% CIs of each condition are shown. # Marginally significant difference (*p* < 0.1, but *p* > 0.05).

The CSE data (Figure 3A) revealed no effect of groups (F_(1,18)_ = 0.156, *p* = 0.697, ${ƞ}_{p}^{2}$ = 0.009), no effect of sessions (F_(1,18)_ = 0.085, *p* = 0.774, ${ƞ}_{p}^{2}$ = 0.005), and no session–group interaction (F_(1,18)_ = 0.156, *p* = 0.697, ${ƞ}_{p}^{2}$ = 0.009). This shows that CSE did not differ between the high- and low-flavanol beverages, suggesting no chronic change in corticospinal excitability.

The CSP data (Figure 3B) revealed no effect of groups (F_(1,18)_ = 0.120, *p* = 0.733, ${ƞ}_{p}^{2}$ = 0.007), no effect of sessions (F_(1,18)_ = 0.030, *p* = 0.864, ${ƞ}_{p}^{2}$ = 0.002), and no session–group interaction (F_(1,18)_ = 0.120, *p* = 0.733, ${ƞ}_{p}^{2}$ = 0.007). This shows that CSP duration did not differ between the high- and low-flavanol beverages, suggesting no chronic change in corticospinal inhibition.

#### 3.2.2. No Chronic Effects of Cocoa Flavanols on Intracortical Facilitation and Inhibition

The SICF data (Figure 3C) revealed no effect of groups (F_(1,18)_ = 0.002, *p* = 0.969, ${ƞ}_{p}^{2}$ < 0.001), no effect of sessions (F_(1,18)_ = 0.017, *p* = 0.897, ${ƞ}_{p}^{2}$ = 0.001), and no session–group interaction (F_(1,18)_ = 0.002, *p* = 0.969, ${ƞ}_{p}^{2}$ < 0.001). This shows that SICF did not differ between the high- and low-flavanol beverages, suggesting no chronic change in intracortical facilitation.

The SICI data (Figure 3D) revealed a session–group interaction (F_(1,18)_ = 4.147, *p* = 0.057, ${ƞ}_{p}^{2}$ = 0.187). After the chronic intervention, the interaction revealed that SICI decreased in the low-flavanol group (164 ± 69%) compared to the high-flavanol one (82 ± 38%; *p* = 0.057, Cohen’s d = 0.911). However, neither the low- (*p* = 0.204, Cohen’s d = 0.575) nor high-flavanol (*p* = 0.383, Cohen’s d = 0.290) groups differed from the pre-intervention values (100%). This suggests that the post-chronic difference between the groups was driven by SICI decreasing in the low-flavanol group, but increasing in the high-flavanol one. Similarly to the LICI results in the acute intervention, this makes it unclear if the chronic intake of high flavanol robustly altered SICI compared to the low-flavanol group and pre-intervention values.

The ICF data (Figure 3E) revealed no effect of groups (F_(1,18)_ < 0.001, *p* = 0.996, ${ƞ}_{p}^{2}$ < 0.001), no effect of sessions (F_(1,18)_ < 0.001, *p* = 0.999, ${ƞ}_{p}^{2}$ < 0.001), and no session–group interaction (F_(1,18)_ < 0.001, *p* = 0.996, ${ƞ}_{p}^{2}$ < 0.001). This shows that ICF did not differ between the high- and low-flavanol beverages, suggesting no chronic change in intracortical facilitation.

The LICI data (Figure 3F) revealed no effect of groups (F_(1,18)_ = 2.168, *p* = 0.158, ${ƞ}_{p}^{2}$ = 0.108), no effect of sessions (F_(1,18)_ = 0.009, *p* = 0.925, ${ƞ}_{p}^{2}$ = 0.001), and no session–group interaction (F_(1,18)_ = 2.168, *p* = 0.158, ${ƞ}_{p}^{2}$ = 0.108). This shows that LICI did not differ between the high- and low-flavanol beverages, suggesting no chronic change in intracortical inhibition.

#### 3.2.3. Estimated Effect Sizes to Power Future Studies

To power future studies seeking to replicate the present results [81,82], pairwise effect sizes and their 95% CIs of each comparison of the acute and chronic interventions are reported in Table 2. Note that comparisons were within-subject in the acute (*n* = 20) and between-subject in the chronic intervention (2 groups of *n* = 10). As such, Cohen’s dz and Cohen’s d effect sizes are reported. Overall, the results suggest that cocoa flavanols do not meaningfully alter brain excitability in young healthy adults.

For the acute intervention, the results revealed that the average effect sizes were negligible to modest for the acute intervention (absolute Cohen’s dz between 0.1 and 0.5 ± ~0.4), suggesting that cocoa flavanols did not importantly alter brain excitability. Reinvesting these results, an a priori analysis using G*Power (v.3.1.9.4) revealed that future studies should recruit at least 34 participants to detect a significant difference (*p* < 0.05) at a Cohen’s dz value of 0.5 (assuming 80% power and a two-tailed dependent *t*-test). The required number of participants will further increase if smaller effects are investigated. For instance, assuming the same parameters as above, detecting a significant difference at a Cohen’s dz value of 0.2 would require ~200 participants. Finally, these estimations may vary, as the 95% CIs were ±~0.4, suggesting that the “true” effect sizes could range from very negligible (<0.1) to above large (>0.9).

For the chronic intervention, the results revealed that effect sizes greatly varied between negligible to above large values (absolute Cohen’s d values between ~0.0 and 0.9), which is likely due to the present limited sample size for between-subject comparisons. As above, future studies reinvesting these results to investigate the chronic effects of cocoa flavanols on brain excitability should recruit at least 2 groups of 26 participants each (*n* = 52) to detect a Cohen’s d value of 0.8 (assuming 80% power and a two-tailed independent *t*-test). As for the acute intervention, the number of participants will increase if smaller effects are investigated. For instance, detecting significant differences at a Cohen’s d value of 0.5 would require 2 groups of 64 participants each (*n* = 128), and detecting significant differences at a Cohen’s d value of 0.2 would require 2 groups of ~400 participants. Finally, these estimations may importantly vary, as the 95% CIs were ~0.9, suggesting that the “true” effect sizes could range from very negligible (Cohen’s d of <0.1) to very large (Cohen’s d of >1.7). Overall, large-scale studies will be required to ascertain the size of both the acute and chronic effects of flavanols on brain excitability.

## 4. Discussion

This work used TMS to test the hypothesis that ingesting cocoa flavanols would alter brain excitability in humans. Specifically, the effects of acute and 5-day chronic cocoa flavanol interventions on excitability were assessed in young healthy adults. Overall, the results do not support that cocoa flavanols acutely or chronically alter corticospinal or intracortical excitability. Specifically, the results from both the acute and chronic interventions showed no robust change in CSE, CSP duration, SICF, SICI, ICF, or LICI upon ingesting the high-flavanol beverage compared to the low-flavanol one *and* pre-intervention (baseline) values. Moreover, the effect sizes of the acute intervention revealed negligible to modest values (absolute Cohen’s dz values between ~0.1 and 0.5), suggesting that the acute effects of cocoa flavanols on brain excitability are modest at best. The effect sizes of the chronic intervention varied greatly (absolute Cohen’s d values between ~0.0 and 0.9), which should be interpreted with caution because of the limited sample sizes of the two groups (*n* = 10). Overall, one possibility is that cocoa flavanols do not yield a net change in brain excitability in humans. Future studies should take into account the relatively small effects of the present results, and investigate if cocoa flavanols would be more effective at altering brain excitability in older adults [83] or in clinical populations [84] than in younger adults.

### 4.1. Acute Intervention: No Robust Effect of Cocoa Flavanols on Brain Excitability

One important result is that cocoa flavanols did not alter CSE, CSP duration, SICF, SICI, or ICF at 1 and 2 h after oral ingestion, suggesting no acute alteration in corticospinal (CSE, CSP duration) or intracortical excitability (SICF, SICI, ICF). At face value, this null result suggests that cocoa flavanols yield no net effect on the two major ionotropic neurotransmission systems—glutamate and GABA_A_—within the central nervous system [73]. However, although previous in vitro and vivo evidence suggests that flavonoids acutely cross the BBB [24,25,26,27,28,29], whether flavanols acutely cross the BBB in humans remains unclear [31]. As such, one interpretation is that the present null results are due to poor (or absent) flavanol bioavailability in the human brain. It may also be that the cocoa flavanol-induced NO release [43,44,45,46,47] has too short a half-life [85,86] to acutely alter brain excitability. Nonetheless, the present null results align with previous human work showing that cocoa flavanols acutely enhance neurovascular coupling but do not alter brain activity as assessed with electroencephalography in healthy adults [10], suggesting that the effects of flavanols do not acutely permeate beyond the cerebral endothelium in the human brain. An alternative interpretation is that flavanols acutely cross the BBB, but would predominantly alter brain excitability in older adults or clinical populations showing deficits in brain energy regulation and oxygen metabolism [83,87]. In support, although cocoa flavanols enhance cognition in young healthy adults [88], evidence has shown that such cognitive improvements are more pronounced in older adults [83,89,90]. This suggests that cocoa flavanols show a ceiling effect in their ability to acutely alter brain functions such as brain excitability in young healthy adults, which could explain the present null results. Whether future studies would support this possibility remains to be determined.

A noteworthy result is that cocoa flavanols altered LICI (Figure 2F), suggesting that flavanols acutely modulated type B (metabotropic) GABA (GABA_B_)-mediated inhibition [73]. If replicated by future studies, one implication would be that cocoa flavanols acutely alter at least some components of brain excitability. Moreover, this result would resonate with the possibility that cocoa flavanol-induced vasodilation [43,44,45,46,47] causes mechanical forces that alter brain excitability [60] through GABA_B_ receptors [61]. Although intriguing, the results, however, did not reveal a clear effect of ingesting high levels of cocoa flavanols on LICI. Namely, on the one hand, the results revealed that the low-flavanol control beverage decreased LICI compared to the high-flavanol one. These results suggest that LICI did not change in response to the high-flavanol beverage and that this difference was driven by a decrease in GABA_B_-mediated inhibition in the low-flavanol control condition. Although the exact causes driving this decrease in LICI remain unclear, the high- and low-flavanol beverages were matched for their caffeine and theobromine content (Table 1), suggesting that differences in methylxanthine content cannot account for this result (see [91] for support). On the other hand, neither of the beverages altered LICI compared to the pre-intervention baseline values, which suggests that the acute consumption of high (or low) levels of cocoa flavanols does not robustly alter GABA_B_-mediated inhibition. To ascertain the potential effects of cocoa flavanols on GABAergic inhibition, future studies could use additional techniques, such as electroencephalography paired with TMS [92].

### 4.2. Chronic Intervention: No Robust Effect of Cocoa Flavanols on Brain Excitability

The chronic effects of consuming one high- or low-flavanol beverage per day for 5 consecutive days on brain excitability were also explored in the current study. For the acute intervention, the results revealed no effect of cocoa flavanols on CSE, CSP duration, SICF, ICF, or LICI, suggesting no chronic alteration in corticospinal or intracortical excitability. One possibility is that flavanols must be ingested over a period longer than 5 consecutive days before they accumulate to significant levels in the brain [17,18,19,20,21,22,23], suggesting that the present chronic intervention was insufficiently long to alter brain excitability. To ascertain this, future studies could prolong the present chronic intervention over several weeks, as consuming high doses of cocoa flavanols for up to 12 weeks is safe for healthy human adults [93]. However, this possibility would not account for the result that SICI was altered after the chronic intervention (Post Chronic; Figure 3D), suggesting that chronic cocoa flavanol consumption altered GABA_A_-mediated inhibition. On the one hand, as with LICI in the acute intervention, the results revealed that the control low-flavanol beverage decreased SICI compared to the high-flavanol one. This suggests that high doses of cocoa flavanols did not alter SICI and that this difference was driven by a decrease in GABA_A_-mediated inhibition in the control low-flavanol group. Despite this, an effect of cocoa flavanols of SICI would align with their reported capacity to act as ligands to GABA_A_ receptors [39,94,95], presumably explaining the therapeutic potential of flavonoids to treat depression and anxiety disorders [96], as well as epilepsy [97,98]. If this result were ascertained by future studies, one implication would be that flavanols alter intracortical GABA_A_-mediated inhibition when consumed chronically, but not acutely. However, on the other hand, SICI did not differ from the pre-intervention baseline values (post- vs. pre-chronic; Figure 3D), which does not provide clear evidence that the present chronic consumption of high levels of cocoa flavanols robustly altered GABA_A_-mediated inhibition. As a result, one conservative interpretation is that 5-day chronic consumption of cocoa flavanols does not robustly alter corticospinal or intracortical excitability. However, because of their potential therapeutic applications [96,97,98], the possibility that cocoa flavanols alter GABA_A_-mediated inhibition in humans warrants future investigations with larger samples and/or populations who may be more susceptible to the benefits of dietary flavanols [4,83,87].

### 4.3. Limitations

First, although the present doses of cocoa flavanols are at the higher end of the spectrum of those used in previous work [45,46,65,66], they were not adjusted to the height/weight and sex of participants. One possibility is that systemic cocoa flavanol concentrations must be individualised to robustly alter brain excitability (see [99,100]). Second, participants were asked to maintain their dietary habits, exercise levels and caffeine consumption 24 h prior to the sessions of both the acute and chronic interventions, which could presumably have confounded the present results. Here, it was reasoned that any meaningful effect of flavanol on brain excitability should be apparent regardless of participants’ daily habits for the results to yield generalisable implications in real-life settings. Whether the acute and chronic effects of cocoa flavanols on brain excitability would be more important in adults with lower diet quality and habitual flavanol consumption remains to be determined (see [4]). Moreover, as participants’ diet was not measured during the chronic intervention, whether within- and between-subject differences in energy intake over 5 days (see [101,102,103]) would alter brain excitability responses to cocoa flavanols remains unknown. This should be addressed by future studies. Third, the a priori power calculation revealed that the acute intervention was appropriately powered to detect a large effect (Cohen’s dz of 0.8), here defined as the smallest effect of interest. However, two groups of 10 were used to explore the chronic effects of cocoa flavanols, which limited statistical power. Future work should use larger samples to replicate the present results. Fourth, the present variables are indirect assays of intracortical glutamatergic activity, as well as GABA_A_- and GABA_B_-mediated inhibition [73]. Although this work suggests that cocoa flavanols have no net effect on these neurotransmission systems, a remaining possibility is that flavanols would alter other major neuromodulator systems, such as dopamine [104] and serotonin [105]. Future work should address this possibility.

## 5. Conclusions

This work shows that compared to a control low-flavanol beverage (5 mg), ingesting beverages containing high levels of cocoa flavanols (695 mg) does not acutely (up to 2 h after ingestion) or chronically (1 beverage a day for 5 consecutive days) alter corticospinal or intracortical excitability in young healthy adults. One possibility is that cocoa flavanols predominantly alter neurovascular coupling but yield no net effect on brain excitability in young healthy adults. Future studies should increase intervention durations to maximize the acute and chronic accumulation of flavanols in the brain and further investigate if cocoa flavanols are more effective at altering brain excitability in ageing and clinical populations.

## Figures and Tables

**Table 1:** Composition of a single 12 g cocoa powder intervention.

	High Flavanol	Low Flavanol
Total polyphenols (mg)	1246.8	260.0
Total flavanols (mg)	695.0	5.6
Procyanidins (dimers-decamers; mg)	459.6	ND
(−)-Epicatechin (mg)	150.0	<6
(−) and (+)-Catechin (mg)	85.4	<6
Theobromine (mg)	262.8	278.4
Caffeine (mg)	27.6	22.2
Fat (g)	1.7	1.3
Carbohydrates (g)	2.7	1.2
Protein (g)	2.7	2.7
Fibre (g)	1.8	4.0
Energy (kcal)	41.4	36.6

The values in this table are valid for both the acute and chronic interventions. In this table, “mg” means milligrams, “g” means grams, and “kcal” means kilocalorie.

**Table 2:** Pairwise effect sizes (point estimate ± 95% CIs) for the acute and chronic interventions.

**High- vs. Low-Flavanol Beverages**						
	**CSE**	**CSP**	**SICF**	**SICI**	**ICF**	**LICI**
Post1	0.112 ± 0.438	0.341 ± 0.447	0.277 ± 0.443	0.263 ± 0.443	−0.051 ± 0.338	−0.329 ± 0.455
Post2	0.399 ± 0.451	0.089 ± 0.438	−0.117 ± 0.441	−0.152 ± 0.443	−0.397 ± 0.461	−0.432 ± 0.464
Combined	0.292 ± 0.444	0.259 ± 0.443	0.082 ± 0.438	0.146 ± 0.439	−0.242 ± 0.448	−0.494 ± 0.471
**High- vs. Low-Flavanol Groups**						
	**CSE**	**CSP**	**SICF**	**SICI**	**ICF**	**LICI**
Post-chronic	−0.177 ± 0.885	−0.155 ± 0.884	0.018 ± 0.876	−0.911 ± 0.990	−0.002 ± 0.877	0.658 ± 0.910

Positive effect size values mean that high flavanol is greater than low flavanol. Conversely, negative effect size values mean that low flavanol is greater than high flavanol. The “Combined” row reports the effect sizes of the marginal mean from the (combined) Post1 and Post2. In this table, “CSE” means corticospinal excitability, “CSP” means corticospinal silent period, “SICF” means short intracortical facilitation, “SICI” means short intracortical inhibition, “ICF” means intracortical facilitation and “LICI” means long intracortical inhibition. “Post1” and “Post2” refer to the time points following beverage ingestion in the acute intervention. “CIs” means confidence intervals.

## Data Availability

This work’s data are freely available at the following URL: https://docs.google.com/spreadsheets/d/1CRet2sgkiFUhy5mPInBCM7EIXqCB_fgq/edit?usp=sharing&ouid=115650053125584404489&rtpof=true&sd=true (accessed on 15 January 2024).
